# Is a cure for HIV feasible? A proof-of-concept in humanized mice

**DOI:** 10.1186/1742-4690-10-S1-O40

**Published:** 2013-09-19

**Authors:** Roberto Speck

**Affiliations:** 1 University Hospital of Zurich, Switzerland

## 

Three strategies are being pursued to cure HIV: therapeutic vaccination, eradication of the latent reservoir, and gene engineering an HIV resistant immune system. Each strategy must overcome major hurdles. The very small number of latently infected cells in HIV-infected patients and the lack of a small-animal model are major challenges for the study of the latent HIV reservoir. Similarly, testing a gene-engineered HIV-resistant immune system *in vivo* was hampered by the lack of an animal model. Humanized mice are generated by transplanting human CD34+ cells into immunocompromised mice, which then give rise to a human lymphoid system. These mice are highly susceptible to high-titer HIV infection. Upon ART, HIV RNA is suppressed within 2–4 weeks, and interruption of the therapy results in viral rebound. Advances in gene engineering (e.g., SIN vectors, Crisp) have enabled the manipulation of genes in hemopoetic progenitor cells and, therefore, made feasible the study of transgenes for their effect on HIV in progeny cells. Using data from my laboratory, I will describe the current state of the art for humanized mice for studying eradication of HIV and the gene engineering of an HIV-resistant immune system.

